# Next-generation microbiological testing in intraabdominal infections with PCR technology

**DOI:** 10.1007/s00423-024-03298-9

**Published:** 2024-04-03

**Authors:** Julian Horn, Philipp Höhn, Johanna Strotmann, Britta Majchrzak-Stiller, Marie Buchholz, Waldemar Uhl, Torsten Herzog

**Affiliations:** 1 Department of General and Visceral Surgery, St. Josef-Hospital Bochum, Ruhr University Bochum, Gudrunstraße 56, 44791 Bochum, Germany; 2 Department of General and Visceral Surgery, Division of Molecular and Clinical Research, St. Josef-Hospital Bochum, Ruhr University Bochum, Gudrunstraße 56, 44791 Bochum, Germany; 3 Department of General Surgery and Visceral Surgery, Klinikum Vest, Ruhr University Bochum, Recklinghausen, Germany

**Keywords:** Intraabdominal infection, Resistant microorganisms, PCR testing

## Abstract

**Purpose:**

Intraabdominal infections (IAI) are increasing worldwide and are a major contributor to morbidity and mortality. Among IAI, the number of multi-drug resistant organisms (MDRO) is increasing globally. We tested the Unyvero A50® for intraabdominal infections, compared the detected microorganisms and antibiotic resistance, and compared the results with those of routine microbiology.

**Methods:**

We prospectively compared samples obtained from surgical patients using PCR-based Unyvero IAI cartridges against routine microbiology for the detection of microorganisms. Additionally, we identified clinical parameters that correlated with the microbiological findings. Data were analyzed using the t-test and Mann–Whitney *U* test.

**Results:**

Sixty-two samples were analyzed. The PCR system identified more microorganisms, mostly Bacteroides species, *Escherichia coli*, and *Enterococcus* spp. For bacterial resistance, the PCR system results were fully concordant with those of routine microbiology, resulting in a sensitivity, specificity, and positive and negative predictive value (PPV, NPV) of 100%. The sensitivity, specificity, PPV, and NPV for the detection of microorganisms were 74%, 58%, 60%, and 72%, respectively. CRP levels were significantly higher in patients with detectable microorganisms. We identified more microorganisms and bacterial resistance in hospital-acquired intra-abdominal infections by using the PCR system.

**Discussion:**

IAI warrants early identification of the microorganisms involved and their resistance to allow for adequate antibiotic therapy. PCR systems enable physicians to rapidly adjust their antibiotic treatment. Conventional microbiological culture and testing remain essential for determining the minimal growth inhibition concentrations for antibiotic therapy.

## Introduction

Intraabdominal infections (IAI) are a huge burden on healthcare systems worldwide. IAI have a higher morbidity and mortality if management is poor [1]. IAIs are usually classified into uncomplicated and complicated IAI and into community- and hospital-acquired IAIs. The frequency of resistant microorganisms is low in uncomplicated, and community acquired IAIs, while complicated and hospital acquired IAIs show a higher rate of resistant microorganisms [2].

### Treatment

Intraabdominal infection therapy includes source control, intensive care management, and antibiotic therapy. Source control is either surgical treatment or interventional percutaneous drainage [3]. The least invasive approach that achieves adequate source control is recommended, especially in critically ill patients, who might be too unstable for surgical procedures [2, 3]. Intensive care treatment options have improved over the last decade, enabling physicians to stabilize patients before and after definitive treatment.

Antibiotic therapy must cover all the microorganisms involved. Broad-spectrum therapy is often indispensable in critically ill patients, especially when multidrug-resistant organisms (MDRO) are suspected. Local and individual risk factors for MDRO, e.g., prior antibiotic treatment, former ICU treatment, age over 60 years, male sex, and previous length of hospital stay, should be considered for antibiotic therapy [4]. Broad-spectrum-antibiotic treatment, however, promotes the emergence of more resistance among the involved bacteria. Precise use of antibiotics minimizes the development of resistance in microorganisms. PCR-based analysis would enable quick identification of the microorganisms involved and their antibiotic resistance for earlier adjustment of antibiotic treatment.

### Involved microorganisms

The microorganisms involved primarily originate in the gastrointestinal tract. The upper gastrointestinal tract is less densely populated with Gram-positive and Gram-negative aerobic and facultative anaerobic organisms. The lower gastrointestinal tract is densely populated with facultative and obligate anaerobic organisms. [5] Aerobic bacteria include *E. coli*, *Enterococci* and other Enterobacteriaceae, and anaerobic bacteria include the *Bacteroides fragilis* group and clostridia [6].

### Consequences of emerging bacterial resistances

Highest rates of antibiotic resistances are found in *E. coli, S. aureus, K. pneumoniae, S. pneumoniae, A. baumannii, P. aeruginosa*, and *Enterococcus* spp. [7]. Extended spectrum beta-lactamases (ESBL) and carbapenemases have the greatest impact on morbidity, mortality, length of stay (LOS), and increasing costs in healthcare systems. Current studies estimate 5 million annual deaths worldwide due to antimicrobial resistance [7]. Involved resistant microorganisms are usually treated with broad-spectrum antibiotics; however, broad-spectrum antibiotic treatment also increases the development of new antibiotic resistance.

### Diagnostic

Routine microbiological testing consists primarily of bacterial culture in different media and exposure to antibiotics to check for growth inhibition. Bacteria were grown on different agar plates and identified by phenotype. The samples were obtained as fluids, swabs, or tissue samples. Some generalized information is available within a few hours, but full identification usually takes more than 48 h. Resistance testing took up to 24 h. Up to 72 h can be passed until full results are available.

## Methods

The samples were obtained from the surgical department at St. Josef Hospital Bochum during operations or percutaneous drainage. Samples were split for separate analyses using the Unyvero system and routine microbiology. Microbiological testing was performed at the Institute for Hygiene and Microbiology, Department of Medical Microbiology, Ruhr University, Bochum. Tests using the Unyvero system were performed at the Division of Molecular and Clinical Research, St. Josef Hospital, Ruhr University, Bochum, in accordance with the supplier’s instructions. Probes were lysed for 30 min in L4 Lysater® to expose the DNA and assembled with the IAI cartridge containing the Master Mix tube®. DNA was amplified using an A50 Analyzer ® within 4 h. Amplified DNA was detected using array hybridization. Samples were collected from patients with peritonitis, intra-abdominal abscesses, necrotizing pancreatitis, and infected pancreatic or biliary fistulas.

Sixty-two samples were eligible for analysis. Tests were performed immediately, and the samples were stored at minus twenty degree Celsius. Clinical data were extracted from patient records.

### Unyvero IAI testing

The IAI panel detects the following microorganisms: *Acinetobacter baumannii* complex, *Aeromonas* spp., *Bacteroides fragilis* group, *Candida albicans*, *Candida glabrata*, *Candida* spp., *Candida tropicalis*, *Candida krusei, Clostridioides difficile, Clostridium perfringens*, coagulase-negative staphylococci, *Cutibacterium acnes*, *Klebsiella aerogenes*, *Enterobacter cloacae* complex, *Enterococcus* spp., *Enterococcus faecalis, Escherichia coli, Finegoldia magna, Klebsiella oxytoca, Klebsiella pneumoniae, Klebsiella variicola, Prevotella* spp., *Bacteroides* spp./*Prevotella* spp., *Proteus* spp., *Pseudomonas aeruginosa*, *Staphylococcus aureus*, and *Streptococcus* spp. The assay at that time contained a universal bacterial analyte for the detection of bacteria not covered by the list above, which was removed in the current version of the Unyvero IAI Application. The following resistance and toxin markers are shown in the panel: aacA4, ctx-M, fosA3, imp, mcr-1, ndm, nimA, nimB, kpc, mecA, mecC, oxa-23, oxa-24/40, oxa-48, oxa-58, qnrA, qnrB, qnrS, tetA, vanA, vanB, vim, tcdB, and stx1/2.

### Statistical analysis

The results were compared, and PCR results were assumed to be correct when they were in accordance with results from conventional microbiology. Overreporting and underreporting were considered false results. Weighted sensitivity was calculated as 100 × TP (TP + FP). Weighted specificity was calculated as 100 × TN (FP + TN). The positive predictive value (PPV) was calculated as 100 × TP (TP + FP). The negative predictive values were calculated as 100 × TN (FN + TN).

Further analysis included parameters for positive or negative correlation for the detection of microorganisms and polymicrobial infection using the Unyvero system. The normal distribution of metric variables was tested using the Shapiro–Wilk test. If the distribution of the variables was normal (p = ^3^ 0.1), the results were analyzed using the t-test (for more than two groups, the f-test was used). If there was no normal distribution, the data were analyzed using the Mann–Whitney *U*-test (for more than 2 groups, Kruskal-Willis test was used). Frequency distribution in categorical variables was analyzed with chi^2^-test. Tables 3, 4, 5, 6 and 7 present the results.

## Results

**Fig. 1:**
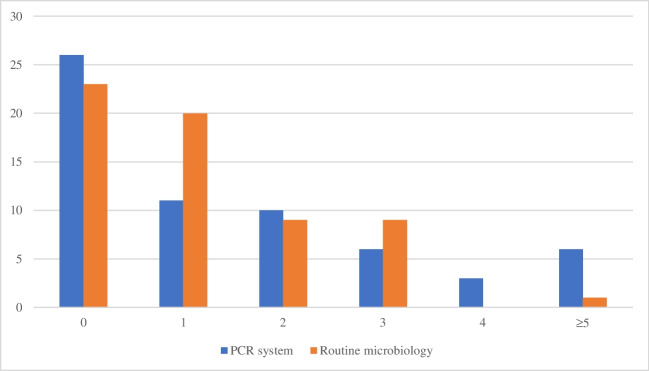
Number of detected microorganisms per sample. Legend: *X*-axis shows the number of pathogens per sample; *Y*-axis shows the number of samples

We analyzed 62 samples from patients undergoing treatment for IAIs. The PCR system detected a total of 95 (overlap of *Bacteroides* species may led to microorganisms being counted twice; for further information, see Appendix) microorganisms; routine microbiology found a total of 66 bacteria. The PCR system detected significant more microorganisms in our samples, namely 2.64 microorganisms per positive sample, in comparison to 1.83 microorganisms per positive sample in routine microbiology (*p*-value: 0.007). The PCR system detected more polymicrobial infections with ≥ 4 microorganisms. Routine microbiological analysis revealed more monomicrobial infections. The Unyvero system and routine microbiology detected the negative samples equally well. In 42% (26/62) of samples with the Unyvero system and 37% (23/62) of samples with routine microbiology, no microorganisms were detected. The number of pathogens per sample is shown in Fig. 1. The distributions of the identified microorganisms are listed in Table 1.

**Table 1:** Distribution of detected microorganisms

Detected microorganisms	Unyvero system	Routine microbiology
Bacteroides/*Prevotella* species	14	5
Issatchenkia orientalis	0	0
*Clostridium perfringens*	2	0
*Finegoldia magna*	1	0
*Klebsiella pneumoniae*	5	5
*Propionibacterium acnes*	0	0
*Aeromonas* species	0	0
*Enterococcus* species	16	3
*Bacteroides fragilis* group	12	4
*Staphylococcus aureus*	1	1
*Acinetobacter baumannii* complex	0	0
*Klebsiella variicola*	0	0
*Enterobacter aerogenes*	0	0
*Klebsiella oxytoca*	3	2
*Proteus* species	3	2
*Pseudomonas aeruginosa*	3	3
*Clostridium difficile*	2	0
*Coagulase negative staphylococci*	3	3
*Enterococcus faecalis*	2	4
*Streptococcus* species	6	8
*Enterobacter cloacae* complex	1	2
*Escherichia coli*	13	13
*Candida* species	4	0
*Candida albicans*	2	7
*Candida glabrata*	0	0
*Candida tropicalis*	0	0
Universal bacteria	2	-

Legend: Distribution of microorganisms with the Unyvero system and in routine microbiology

The Unyvero system detected 11 resistance genes. These results matched the resistance from routine microbiology. The sensitivity, specificity, PPV, and NPV of this analysis were 100%. The results are presented in Table 2.

**Table 2:** Detected resistance genes in the PCR analysis and antibiotic resistances in routine microbiology

Resistance gene in PCR analysis	Number of detections	Resistance in routine microbiology
aac4A	2	2
CTX-M	1	1
qnrB	1	1
tetA	1	1
mecA	4	4
vanB	2	2

Legend: Detected resistance genes in with the PCR system in comparison to antibiotic resistance in routine microbiology

Using conventional microbiology as the gold standard, PCR probes had a sensitivity of 74%, specificity of 58%, positive predictive value (PPV) of 60%, and negative predictive value (NPV) of 72%.

**Fig. 2:**
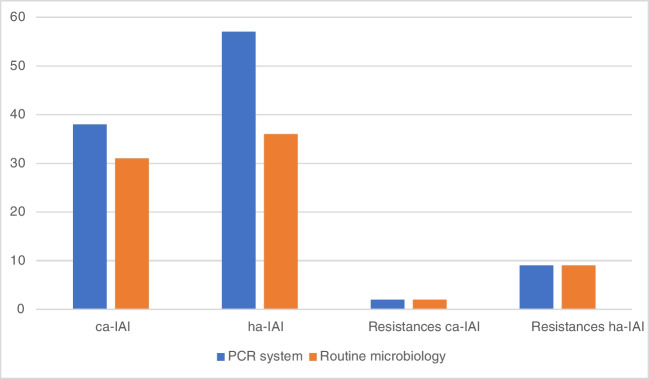
Comparison between detected microorganisms and resistances in ca-IAI and ha-IAI with the PCR system and routine microbiology. Legend: *ca-IAI* community acquired intraabdominal infections, *ha-IAI* hospital acquired intraabdominal infections

We analyzed 36 samples from community-acquired intra-abdominal infections (caIAIs) and 26 from hospital acquired intraabdominal infections (haIAIs). There were significantly more microorganisms in haIAI with the PCR system (*p* value: 0.007). We detected 57 microorganisms in haIAI (2.19 per sample) vs. 38 microorganisms in caIAI (1.05 per sample). We found 36 microorganisms in haIAI (1.39 per sample) vs. 31 pathogens (0.86 per sample) using routine microbiology (*p* value: 0.052). There were nine resistance genes in haIAI (four mecA, two vanB, two aacAc4, and one CTX-M) and two in caIAI (TetA and qnrb) (*p* value: 0.007) (Fig. 2).

We analyzed the influence of CRP levels, white blood cell (WBC) count, preoperative antibiotic treatment, and the origin of the IAI on the presence of microorganisms in the PCR system and routine microbiology.

The CRP levels were significantly higher in patients with positive samples than in those without detected microorganisms in the PCR analysis. Routine microbiology also showed significantly elevated CRP levels in positive samples. For the WBC count, no significant association was demonstrated either with the PCR System or in routine microbiology. The results are presented in Table 3.

**Table 3:** Correlation between positive tests in the PCR-system and routine microbiology for CRP levels and white blood cell count (WBC)

	Variable	Positive result	*N*	mean	SD	Min	Max	p^1)^
PCR system	CRP	Yes	36	144.36	99.29	5.0	341.5	< 0.001
		No	26	48.97	65.45	5.0	262.2	
	WBC	Yes	36	14,376	7639.2	3040	44,900	0.516
		No	26	16,219	19,593	3580	109,030	
Routine microbiology	CRP	Yes	39	133.69	101.12	5.0	341.5	0.002
		No	23	54.7	66.1	5.0	262	
	WBC	Yes	39	13,958	7409	3040	44,900	0.920
		No	23	17,574	20,217	3580	109,030	

Legend: *N* number, *mean* mean value, *SD* standard deviation, *Min* minimum, *Max* maximum, ^*1)*^*p* value of the Mann–Whitney *U*-test, *CRP* mg/l, *WBC* per µl

We analyzed the rate of positive results according to the origin of IAI. There were more positive results with the PCR system in IAI from the lower gastrointestinal tract 83% (10/12) and 52% (25/50) of other origins of IAIs (*p* value: 0.048). The results are presented in Table 4.

**Table 4:** Correlation between positive test for microorganisms in the PCR test system and routine microbiology

		PCR system					Routine microbiology				
		Positive result* n* = 36		Negative result *n* = 26			Positive result *n* = 39		Negative result *n* = 23		
		*n*	%	*n*	%	*p*^1)^	*N*	%	*n*	%	*p*^1)^
Origin of intraabdominal infection	Hepatobiliary	10	56	8	44	0.79	11	61	7	39	0.76
	Pancreatic	6	38	10	62	0.23	8	50	8	50	0.22
	LGT	10	83	2	17	0.04	10	83	2	17	0.10
	UGI	9	69	4	31	0.35	9	69	4	31	0.59
	Other	1	33	2	67	–	1	33	2	67	–

Legend: *UGI* upper gastrointestinal tract, *LGT* lower upper gastrointestinal tract, ^1)^*p* value of Chi-quadrat test

We analyzed preoperative antibiotic treatment for positive microbiological diagnostic results. Preoperative antibiotic therapy had no significant correlation with microorganisms detected in the PCR system 53% (20/36) vs. 47% (16/36) (*p* value:0,275). In routine microbiology, no significant difference in preoperative antibiotic treatment was observed. The results are presented in Table 5.

**Table 5:** Correlation between positive test for preoperative antibiotic treatment in the PCR system and routine microbiology

		PCR system					Routine microbiology				
		Positive result *n* = 36	%	Negative result *n* = 26	%	*p*^1)^	Positive result *n* = 39	%	Negative result *n* = 23	%	*p*^1)^
Preoperative antibiotic treatment	No	20	53	18	47	0.275	22	58	16	42	0.304
	Yes	16	67	8	33		17	71	7	29	

Legend: positive, samples with detected microorganisms; negative, samples without detected microorganisms, ^1)^*p* value of Chi-quadrat test; further information on antibiotics, see Appendix

We analyzed the influence of the number of microorganisms on the CRP level, WBC count, origin of IAI, and preoperative antibiotic treatment. There was no significant difference in CRP levels for polymicrobial IAI.

We analyzed the difference in CRP level and WBC count between monomicrobial and polymicrobial infections using PCR-based analysis and conventional microbiology. No significant difference was present for CRP level (171.86 ± 104.14 mg/l vs 132.27 ± 92.47 mg/l, *p* value: 0.262) or for WBC count (13,026 ± 5216/µl vs 14,970 ± 8281/µl, *p* value: 0.653) in the PCR system. In routine microbiology, no significant difference in CRP level (135.17/- 103.64 vs 132.08 ± 98.31, *p* value: 0.865) or WBC count (14,938 ± 8520 vs 12,926 ± 5847, *p* value: 0.660) was present. The results are presented in Table 6.

**Table 6:** Correlation between positive tests for CRP levels and white blood cell count (WBC) in polymicrobial IAI with the PCR system and routine microbiology

	Variable	Microorganisms	*N*	Mean	SD	Min	Q1	Median	Q3	Max	*p*^1)^
PCR system	CRP	1	12	171.86	104.14	29.1	74.65	199.9	237.25	341.5	0.262
		≥ 2	24	132.27	92.47	5	44	135.6	180.4	308.3	
	WBC	1	12	13,026	5216	4930	9090	12,880	16,620	23,090	0.653
		≥ 2	24	14,970	8281	3040	9620	14,460	19,800	44,900	
Conventional microbiology	CRP	1	20	135.17	103.64	5	42.3	107.45	219	313.8	0.865
		≥ 2	19	132.08	98.31	5	45.65	135.6	179.7	341.5	
	WBC	1	20	14,938	8520	4930	9440	14,300	17,152	44,900	0.660
		≥ 2	19	12,926	5847	3040	8105	13,070	16,780	23,160	

Legend: *microorganisms* number of microorganisms, *N* number of probes, *mean* mean value, *SD* standard deviation, *Min* minimum, *Q1* lower quartile, *Q3* upper quartile, *max* maximum, ^*1)*^*p* value of the Kruskal–Wallis test, *CRP* mg/l, *WBC* per µl

There was no significant association between polymicrobial infections and the origin of the IAI in the PCR analysis (*p* value: 0.182) or in routine microbiology (*p* value: 0.366). The data are presented in Table 9 in the Appendix.

There was no significant association between polymicrobial infections and preoperative antibiotic treatment in PCR analysis (*p* value: 0.547), or routine microbiology (*p* value: 0.623). The data are presented in Table 10 in the Appendix.

## Discussion

This retrospective study compared the differences between PCR-based analyses and conventional microbiological cultures of microbiological samples from patients with IAIs. Significantly more microorganisms were detected by PCR analysis. In haIAIs, there were more microorganisms with a higher frequency of resistance compared to caIAIs in PCR analyses and conventional microbiological analyses. Among patients with positive culture results, CRP levels were significantly higher in both PCR analyses and conventional cultures.

PCR-based microbiological diagnostic is on the verge to leap into clinical routine. PCR-based essays for specific microorganisms and resistances were developed in the last decade [8]. Earlier studies showed the usability of PCR based testing in intraabdominal infections and SIRS, but focused mostly on blood samples and showed mixed accordance between PCR result and conventional microbiology. [9, 10]. Ciesielczuk et al. proved the usability of PCR based microbiological diagnostic with the Unyvero P50 in a retrospective setting for microorganisms and bacterial resistances using the Unyvero IAI.

The difficulty of growing obligate anaerobic bacteria in routine culture is a well-known problem. Over the last years, an increase in resistances among these microorganisms has been observed [11]. The most common anaerobic microorganisms in our study were from the Bacteroides group, which have the highest rate of antibiotic resistances rates among anaerobic bacteria in the human gut [12]. A defect in the mucosal barrier is necessary for Bacteroides to become pathogenic. If introduced to the bloodstream, a mortality up to 30% is reported in the literature [13]. Anaerobic microorganisms are less likely to be detected in routine microbiological studies. Since PCR-based approaches do not depend on bacterial growth, they usually detect anaerobic bacteria more reliable which can contribute to a higher average number of detected pathogens using PCR-based analysis [14–16].

C-reactive protein (CRP) is a well-established parameter to measure inflammation in humans in clinical practice [17]. Sensitivity and specificity for abdominal pathologies increase with increasing CRP levels in patients with IAIs [18]. Although there are no general cut-off values, elevated CRP levels are associated with bacterial inflammation [19]. The microbiological species do not affect CRP level. In patients with positive culture results, CRP levels were significantly higher in both the PCR and conventional microbiological analyses. White blood cell (WBC) count is routinely used and is elevated in various clinical conditions, including inflammation due to bacterial infections [20, 21]. We did not observe a significant correlation between elevated WBC counts and positive results in the PCR system or routine microbiology. Compared to other biomarkers, a recent randomized controlled trial confirmed CRP to be a robust parameter for differentiating between septic and non-septic patients with systemic inflammatory response syndrome (SIRS) [22]. WBC are more often affected by non-septic causes [23]. To reduce the amount of analysis without detection of microorganisms, we believe that screening patients using routinely available parameters helps identify patients who benefit from fast PCR analysis. We believe that critically ill patients who need rapid effective antibiotic treatment are the primary group who benefit from rapid microbiological analysis. Among the identified WBC, one patient had a very high WBC count. To take this into account, we utilized rank sum-tests (Mann Whitney *U*-test, Kruskal–Wallis test) for statistical analyses.

Hospital-acquired intraabdominal infections are defined as infections starting later than 48 h after hospital admission or as infections in patients with regular contact with health care systems. HaIAIs are associated with change in bacterial spectrum and increased bacterial resistances [24]. We detected significantly more microorganisms and bacterial resistance using the PCR system in patients with haIAIs than in those with community acquired IAIs. These findings reflect the change in bacterial milieu in haIAI [25]. Polymicrobial infections do not necessarily show a worse outcome [26]. Morbidity and mortality are increased in haIAIs [27]. Several studies have shown a three-fold increase in mortality for haIAI compared to caIAIs, mostly because the initial antibiotic therapy is inadequate in 30% of cases [28, 29]. In contrast, immediate and adequate antibiotic therapy in patients with sepsis or septic shock results in decreased mortality [30, 31]. An inappropriate antibiotic therapy is associated with a fivefold reduction of survival in septic shock [32]. From an economic standpoint, earlier adequate therapy can reduce LOS and cost [33–35].

The results of the present study did not show a significant difference between patients with or without preoperative antibiotic treatment, either for the PCR system or for routine microbiology. We expected more positive results in PCR-based analyses than in routine microbiology for patients who received preoperative treatment. Although there is no data for IAIs, studies have demonstrated a loss of pathogen detection in blood cultures under antibiotic treatment [36, 37]. Similar to our results, loss of pathogen detection was reported in a PCR-based analysis of neonatal sepsis [38]. Other studies demonstrated that amplification of dead bacterial DNA is possible with PCR technologies [39]. Potential reasons for inability to amplify DNA could be fast degradation in vivo because of immunological processes and clearance of bacterial remnants after antibiotic exposure. Most patients in our study had received perioperative antibiotics before surgery.

The present study had a heterogeneous distribution of patients who received antibiotic treatment. Patients with IAI of pancreatic or hepatobiliary origin of IAI were usually treated for a longer period with antibiotics before surgery. Patients with IAI originating from the UGI or LGI underwent emergency surgery, and antibiotics were administered immediately before surgery was initiated. Because of this discrepancy, antibiotic treatment might have biased the analysis, resulting in fewer microorganisms in the hepatobiliary and pancreatic IAIs. Further studies are needed to examine the in vivo loss of pathogen detection using PCR-based analysis and to clarify its use in microbiological diagnostics in patients undergoing ongoing antibiotic therapy.

We detected more polymicrobial infections with PCR-based analysis. Since intraabdominal infections are mostly polymicrobial, this is to be expected. This is in line with current literature, as other studies had similar result in PCR based testing in blood samples and stool samples [40, 41]. Since all microorganisms in the sample are reported, interpretating these results is important to differentiate which microorganisms are pathogens and which are commensals. The Unyvero P50 offers a semiquantitative scale on the amount of bacterial DNA in the sample. This can help identify the driving pathogens for the infection. To help identify pathogenic microorganisms in polymicrobial intraabdominal infections, a combination with PCR-based testing in blood cultures should be researched.

Bacterial resistance is increasing worldwide, contributing to morbidity, mortality, and overall costs. Eleven resistance genes were identified in the samples. These results match the resistance found in routine microbiology. With new PCR-based methods, identification takes 4–5 h, and therapy can be adjusted earlier. The detected resistance genes can be assigned to the detected microorganisms. Gene transcription is regulated by proteins which can act as suppressors or activators [42]. To obtain information on the gene transcript, a reverse transcriptase PCR would be needed [43]. Our data show a very high accordance for the detected genes and in vivo resistance in clinical practice. Species-specific bacterial resistance mechanisms like cell wall structure or efflux pumps need to be considered for antibiotic therapy as well [44]. The physician should know phenotypic characteristics of the microorganism species and the resulting antibiotic resistance to enable fast PCR-based identification through PCR analysis. Finally, an important consideration is the inability to determine the minimum inhibitory concentration (MIC) using PCR-based analysis. The MIC is currently the best available parameter for estimating in vivo effectiveness against microorganisms [45]. Further studies are needed to determine whether this aspect has an influence on clinical practice.

### Limitations to our study

Our study had some limitations. This prospective nature resulted in many samples lacking microorganisms. Although this reflects real-world usage, it reduces the power of the analysis regarding polymicrobial IAI and the influence of different groups of antibiotics on PCR-based testing. Other studies also showed unexpected high rate of negative cultures in IAIs and sepsis [46, 47]. Because the results mostly matched the routine culture results, we assumed that these samples were without microorganisms. Because many samples were taken during emergency surgery, transport may have been delayed for further microbiological workup. This could be one of the reasons for the high number of negative samples. In retrospect, we need to examine some indications for microbiological diagnostic. Some samples came from the UGI, where peritonitis could be induced chemically from gastric acid. From a clinical standpoint however, involvement of microorganisms had to be expected. Another explanation could be hard to cultivate obligate anaerobic microorganisms, that are not represented on the Panel of the Unyvero P50. Although rare in intraabdominal infection, viruses could also lead to negative samples. Prior antibiotic treatment has been discussed as an explanation for culture-negative IAI, but no connection has been seen in prior studies [48]. This is in line with the data in our study.

### Discrepant results

For the calculated sensitivity, specificity, positive predictive value, and negative predictive value, routine microbiology was used as a reference. Other studies show higher rates for the afore named parameters, when validating results with targeted PCR and basic local alignment search tool (BLAST) analysis [16]. We assume that employing additional analysis would resolve the difference partially. Our goal was evaluating the practicability of the Unyvero P50. How these unmatching results will be interpreted, when more PCR based microbiology will be used, requires more studies and data. This could change microbiological diagnostic drastically. Another factor that we need to consider is the work flow in our institution. While PCR-based analysis was done at our hospital, samples were transported to the Institute for Hygiene and Microbiology, Department of Medical Microbiology, Ruhr University, Bochum, for routine microbiology. We cannot rule out a change in bacterial milieu during time of transport, although the laboratory is in proximity.

In clinical practice, both analytical methods should be used to achieve optimal treatment results, especially for patients with haIAIs, where a higher number of different microorganisms and resistance are common. However, the gold standard remains bacterial culture, especially for the detection of minimal inhibitory concentrations.

## Conclusion

PCR-based systems are making their way to microbiological diagnostics and are here to stay. PCR-based systems offer rapid identification of the most common pathogens with their resistance and allow an early adjustment of antibiotic therapy, and we believe that it is especially beneficial in haIAIs with a high frequency of resistant microorganisms. CRP remains the best predictor of microorganism-related inflammation. Although gene-associated resistances are obtained in PCR-based testing, in vitro testing for the minimum inhibitory concentration is still mandatory to adjust the treatment. Currently, there are advantages in implementing PCR-based testing to achieve a fast turnaround time and guide empirical antibiotic treatment when the resistance gene is present; however, combining both approaches is mandatory. However, we believe that PCR-based systems will significantly influence microbiological diagnostics in the future.

## Data Availability

Data can be obtained on reasonable request.

## Appendix

Please see Table 7, 8, 9 and 10.

**Table 7:** Preoperative antibiotic treatment and detected microorganisms

		Positive PCR result				
		Yes *n* = 36		No *n* = 26		
Variable	Wert	*n*	%	*n*	%	*p* ^1)^
Preoperative antibiotic treatment	Pen	7	87.5	1	12.5	–
	Ceph	1	33.3	2	66.7	–
	Carb	4	66.7	2	33.3	–
	Flour			1	100	–
	Comb	4	80.0	1	20.0	–
	none	20	52.6	18	47.4	–
	other			1	100	–

Legend: *Pen* penicillin, *Ceph* cephalosporins, *Carb* carbapenems, *Fluor* fluoroquinolone, *Comb* combination

**Table 8:** Preoperative antibiotic treatment on number of detected microorganisms in PCR-based analysis

		Number of different microorganisms								
		0 *n* = 26		1 *n* = 12		2 *n* = 12		≥ 3 *n* = 12		
Preoperative Antibiotic treatment	Pen	1	12.5	1	12.5	2	25.0	4	50.0	–
	Ceph	2	66.7					1	33.3	
	Carb	2	33.3	2	33.3	1	16.7	1	16.7	
	Flour	1	100							
	Combination	1	20.0	1	20.0	3	60.0			
	None	18	47.4	8	21.1	6	15.8	6	15.8	
	Other	1	100							

Legend: *Pen* penicillin, *Ceph* cephalosporins, *Carb* carbapenems, *Fluor* fluoroquinolone, *Comb* combination

**Table 9:** Correlation between positive tests for origin of infection and polymicrobial IAI with the PCR system and conventional microbiology

			Number of different microorganisms								
			0 *n* = 26		1 *n* = 12		2 *n* = 12		≥ 3 *n* = 12		
		Wert	*n*	%*	*n*	%*	*n*	%*	*n*	%*	*p* ^1)^
PCR system	Origin of intraabdominal infection	Hepatobiliary	8	44.4	6	33.3	1	5.6	3	16.7	0.182
		Pancreatic	10	62.5	1	6.3	3	18.8	2	12.5	
		LGT	2	16.7	2	16.7	5	41.7	3	25.0	
		UGI	4	30.8	3	23.1	3	23.1	3	23.1	
		Other	2	66.7					1	33.3	
			0* n* = 23		1 *n* = 20		2 *n* = 9		≥ 3 *n* = 10		
		Wert	*n*	%*	*n*	%*	*n*	%*	*n*	%*	*p* ^1)^
Conventional microbiology	Origin of intraabdominal infection	Hepatobiliary	7	38.9	8	44.4	1	5.6	2	11.1	0.366
		Pancreatic	8	50.0	3	18.8	2	12.5	3	18.8	
		LGT	2	16.7	3	25.0	3	25.0	4	33.3	
		UGI	4	30.8	6	24.2	2	15.4	1	7.7	
		other	2	66.7			1			33.3	

Legend: *n* number, *UGI* upper gastrointestinal tract, *LGT* lower upper gastrointestinal tract, ^*1)*^*p* value of Chi-square test

**Table 10:** Correlation between preoperative antibiotic treatment and number of detected pathogens in the PCR system and conventional microbiology

		Number of different microorganisms								
PCR system		0 *n* = 26		1 *n* = 12		2 *n* = 12		≥ 3 *n* = 12		
		*n*	%*	*n*	%*	*n*	%*	*N*	%*	*p* ^1)^
Preoperativ antibiotic treatment	No	18	47.4	8	21.1	6	15.8	6	15.8	0.547
	Yes	8	33.3	4	16.7	6	25.0	6	25.0	
Conventional microbiology		0 *n* = 23		1 *n* = 20		2 *n* = 9		≥ 3 *n* = 10		
		*n*	%*	*n*	%*	*n*	*n*	%*	*n*	
Preoperativ antibiotic treatment	No	16	42.1	12	31.6	4	10.5	6	15.8	0.623
	Yes	7	26.2	8	33.3	5	20.8	4	16.7	

*Legend: n* = *number of samples*

## Abbreviations

IAI: Intraabdominal infections
MDRO: Multidrug-resistant organism
PCR: Polymerase chain reaction
TP: True positive
FP: False positive
TN: True negative
FN: False negative
PPV: Positive predictive value
NPV: Negative predictive value
CRP: C-reactive protein
WBC: White blood cell
LGI: Lower gastrointestinal tract
UGI: Upper gastrointestinal tract
cIAI: Complicated intraabdominal infection
caIAI: Community acquired intraabdominal infection
haIAI: Hospital acquired intraabdominal infection
ESBL: Extended spectrum beta-lactamases
ICU: Intensive care unit
GALT: Gastrointestinal associated lymphatic tissue
SIRS: Systematic inflammatory response syndrome
MIC: Minimum inhibitory concentration
